# *N*-(4-Chloro­phen­yl)-2-hy­droxy­benzamide

**DOI:** 10.1107/S1600536810042030

**Published:** 2010-10-23

**Authors:** Abdul Rauf Raza, Bushra Nisar, M. Nawaz Tahir, Sumaira Shamshad

**Affiliations:** a Department of Chemistry, University of Sargodha, Sargodha, Pakistan; b Department of Physics, University of Sargodha, Sargodha, Pakistan

## Abstract

In the title compound, C_13_H_10_ClNO_2_, the dihedral angle between the aromatic rings is 20.02 (6)° and intra­molecular N—H⋯O and C—H⋯O hydrogen bonds both generate *S*(6) rings. In the crystal, mol­ecules are linked by O—H⋯O hydrogen bonds into *C*(6) chains propagating in [010].

## Related literature

For biological background, see: Samanta *et al.* (2010 ▶). For related structures, see: Raza *et al.* (2009 ▶, 2010*a* ▶,*b* ▶). For graph-set notation, see: Bernstein *et al.* (1995 ▶).
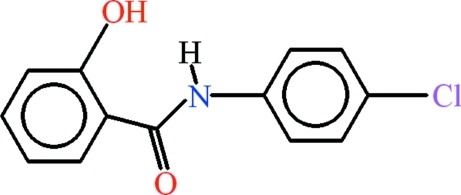


## Experimental

### 

#### Crystal data

C_13_H_10_ClNO_2_
*M* *_r_* = 247.67
Orthorhombic, 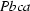

*a* = 7.6832 (3) Å
*b* = 11.0225 (3) Å
*c* = 27.1427 (11) Å
*V* = 2298.66 (14) Å^3^
*Z* = 8
Mo *K*α radiation
μ = 0.32 mm^−1^
*T* = 296 K
0.28 × 0.16 × 0.14 mm

#### Data collection

Bruker Kappa APEXII CCD diffractometer
Absorption correction: multi-scan (*SADABS*; Bruker, 2009 ▶) *T*_min_ = 0.942, *T*_max_ = 0.955
9244 measured reflections
2064 independent reflections
1561 reflections with *I* > 2σ(*I*)
*R*_int_ = 0.027

#### Refinement

*R*[*F* ^2^ > 2σ(*F* ^2^)] = 0.037
*wR*(*F* ^2^) = 0.100
*S* = 1.03
2064 reflections
160 parameters
H atoms treated by a mixture of independent and constrained refinement
Δρ_max_ = 0.18 e Å^−3^
Δρ_min_ = −0.22 e Å^−3^

### 

Data collection: *APEX2* (Bruker, 2009 ▶); cell refinement: *SAINT* (Bruker, 2009 ▶); data reduction: *SAINT*; program(s) used to solve structure: *SHELXS97* (Sheldrick, 2008 ▶); program(s) used to refine structure: *SHELXL97* (Sheldrick, 2008 ▶); molecular graphics: *ORTEP-3* (Farrugia, 1997 ▶) and *PLATON* (Spek, 2009 ▶); software used to prepare material for publication: *WinGX* (Farrugia, 1999 ▶) and *PLATON*.

## Supplementary Material

Crystal structure: contains datablocks global, I. DOI: 10.1107/S1600536810042030/hb5690sup1.cif

Structure factors: contains datablocks I. DOI: 10.1107/S1600536810042030/hb5690Isup2.hkl

Additional supplementary materials:  crystallographic information; 3D view; checkCIF report

## Figures and Tables

**Table 1:** Hydrogen-bond geometry (Å, °)

*D*—H⋯*A*	*D*—H	H⋯*A*	*D*⋯*A*	*D*—H⋯*A*
N1—H1⋯O2	0.84 (2)	1.991 (18)	2.6588 (19)	136.4 (17)
O2—H2⋯O1^i^	0.82 (2)	1.85 (2)	2.6582 (17)	173 (2)
C9—H9⋯O1	0.93	2.31	2.895 (2)	121

Symmetry code: (i) 
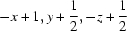
.

**Fig. 1.**
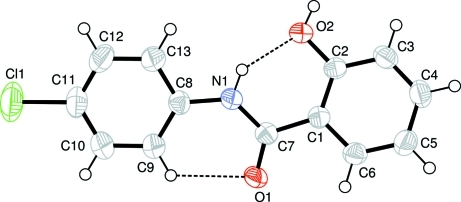
View of the title compound with displacement ellipsoids drawn at the 50% probability level. H-atoms are shown by small circles of arbitrary radius. The dotted lines indicate the intramolecular H-bonds.

**Fig. 2.**
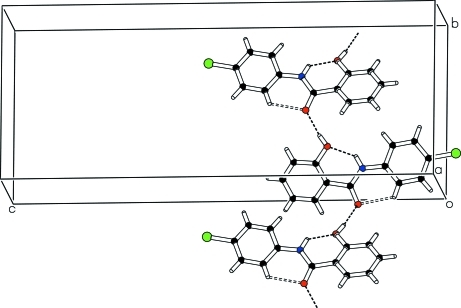
The partial packing for (I), which shows that molecules form one dimensional polymeric chains parallel to b axis.

## supplementary crystallographic information

### Comment

Different synthetic derivatives of benzoxazepine have been reported as anti-tumor and anti-inflammatory agents (Samanta *et al.*, 2010). The title compound (I) was prepared as a precursor for the synthesis of chiral benzoxazepines and it will also be utilized for the complexation with various metals.

We have reported the crystal structures of (II) *i.e.*, 2-hydroxy-5-nitro-*N*-phenylbenzamide (Raza *et al.*, 2010*a*), (III) *i.e.*, 2-Hydroxy-*N*-(3-nitrophenyl)benzamide (Raza *et al.*, 2010*b*) and (IV) *i.e.*, 2-Hydroxy-3-nitro-*N*-phenylbenzamide (Raza *et al.*, 2009) which are related to the title compound.

In (I), the 2-hydroxyphenyl group A (C1–C6/O2) and 4-chloroanilinic group B (C8—C13/N1/CL1) are planar with r. m. s. deviation of 0.0072 and 0.0035 Å, respectively. The dihedral angle between A/B is 20.02 (6)°. There exist intramolecular H-bondings of N—H···O and C—H···O types (Table 1, Fig. 1) completing S(6) ring motifs (Bernstein *et al.*, 1995). The molecules are stabilized in the form of one dimensional polymeric chains extending along the crystallographic *b* axis due to intermolecular H-bondings of O—H···O type (Table 1, Fig. 2).

### Experimental

To a well stirred solution of 2-hydroxy benzoic acid (1.38 g, 0.01 mol, 1 eq) and SOCl_2_ (0.87 ml, 1.42 g, 0.012 mol, 1.2 eq) in dry CHCl_3_, the 4-chloroaniline (1.27 g, 0.01 mol, 1 eq) and Et_3_N (2.08 ml, 1.5 g, 0.015 mol, 1.5 eq) was added slowly at room temperature followed by 3 h reflux. After commencement of reaction, the reaction mixture was cooled to room temperature, neutralized with aqueous NaHCO_3_ (10%) and extracted with EtOAc (3×25 ml). The organic layer was combined, dried over anhydrous Na_2_SO_4_, filtered and concentrated under reduced pressure to afford light yellowish solid. The column chromatographic purification with 0 and 1% EtOAc in petrol (0.5 *L* each) over a silica gel packed column (of 25.5 cm length) afforded colorless prisms of (I) in 24th–106th fraction (10 ml each) upon leaving at room temperature.

### Refinement

The coordinates of H-atoms of amide and hydroxy group were refined. H atoms were positioned geometrically with (C–H = 0.93 Å) and were included in the refinement in the riding model approximation, with *U*_iso_(H) = *xU*_eq_(C, N, O), where *x* = 1.2 for all H-atoms.

### Crystal data

**Table:**

C_13_H_10_ClNO_2_	*F*(000) = 1024
*M**_r_* = 247.67	*D*_x_ = 1.431 Mg m^−^^3^
Orthorhombic, *P**b**c**a*	Mo *K*α radiation, λ = 0.71073 Å
Hall symbol: -P 2ac 2ab	Cell parameters from 1561 reflections
*a* = 7.6832 (3) Å	θ = 3.0–25.3°
*b* = 11.0225 (3) Å	µ = 0.32 mm^−^^1^
*c* = 27.1427 (11) Å	*T* = 296 K
*V* = 2298.66 (14) Å^3^	Needle, colorless
*Z* = 8	0.28 × 0.16 × 0.14 mm

### Data collection

**Table:**

Bruker Kappa APEXII CCD diffractometer	2064 independent reflections
Radiation source: fine-focus sealed tube	1561 reflections with *I* > 2σ(*I*)
graphite	*R*_int_ = 0.027
Detector resolution: 7.5 pixels mm^-1^	θ_max_ = 25.3°, θ_min_ = 3.0°
ω scans	*h* = −9→6
Absorption correction: multi-scan (*SADABS*; Bruker, 2009)	*k* = −13→11
*T*_min_ = 0.942, *T*_max_ = 0.955	*l* = −24→32
9244 measured reflections	

### Refinement

**Table:**

Refinement on *F*^2^	Primary atom site location: structure-invariant direct methods
Least-squares matrix: full	Secondary atom site location: difference Fourier map
*R*[*F*^2^ > 2σ(*F*^2^)] = 0.037	Hydrogen site location: inferred from neighbouring sites
*wR*(*F*^2^) = 0.100	H atoms treated by a mixture of independent and constrained refinement
*S* = 1.03	*w* = 1/[σ^2^(*F*_o_^2^) + (0.0469*P*)^2^ + 0.540*P*] where *P* = (*F*_o_^2^ + 2*F*_c_^2^)/3
2064 reflections	(Δ/σ)_max_ < 0.001
160 parameters	Δρ_max_ = 0.18 e Å^−^^3^
0 restraints	Δρ_min_ = −0.22 e Å^−^^3^

### Special details

**Table:**

Geometry. Bond distances, angles *etc*. have been calculated using the rounded fractional coordinates. All su's are estimated from the variances of the (full) variance-covariance matrix. The cell e.s.d.'s are taken into account in the estimation of distances, angles and torsion angles
Refinement. Refinement of *F*^2^ against ALL reflections. The weighted *R*-factor *wR* and goodness of fit *S* are based on *F*^2^, conventional *R*-factors *R* are based on *F*, with *F* set to zero for negative *F*^2^. The threshold expression of *F*^2^ > σ(*F*^2^) is used only for calculating *R*-factors(gt) *etc*. and is not relevant to the choice of reflections for refinement. *R*-factors based on *F*^2^ are statistically about twice as large as those based on *F*, and *R*- factors based on ALL data will be even larger.

### Fractional atomic coordinates and isotropic or equivalent isotropic displacement parameters (Å^2^)

**Table:**

	*x*	*y*	*z*	*U*_iso_*/*U*_eq_	
Cl1	0.37823 (11)	0.20751 (7)	−0.03285 (2)	0.0959 (3)	
O1	0.33904 (17)	−0.07407 (10)	0.19332 (4)	0.0464 (4)	
O2	0.52554 (18)	0.23574 (11)	0.26024 (5)	0.0482 (5)	
N1	0.3890 (2)	0.12607 (13)	0.18202 (5)	0.0423 (5)	
C1	0.3726 (2)	0.04847 (14)	0.26498 (6)	0.0355 (5)	
C2	0.4477 (2)	0.14916 (14)	0.28844 (6)	0.0373 (6)	
C3	0.4430 (3)	0.15880 (16)	0.33940 (6)	0.0461 (6)	
C4	0.3619 (3)	0.07148 (18)	0.36719 (7)	0.0519 (7)	
C5	0.2852 (3)	−0.02736 (17)	0.34495 (7)	0.0513 (7)	
C6	0.2926 (2)	−0.03846 (15)	0.29454 (6)	0.0421 (6)	
C7	0.3672 (2)	0.02787 (15)	0.21083 (6)	0.0369 (6)	
C8	0.3852 (2)	0.13776 (15)	0.13042 (6)	0.0400 (6)	
C9	0.3543 (3)	0.04338 (18)	0.09804 (7)	0.0552 (7)	
C10	0.3524 (3)	0.0662 (2)	0.04786 (7)	0.0622 (8)	
C11	0.3811 (3)	0.1803 (2)	0.03022 (7)	0.0569 (8)	
C12	0.4125 (3)	0.27414 (19)	0.06207 (8)	0.0622 (8)	
C13	0.4130 (3)	0.25274 (17)	0.11203 (7)	0.0529 (7)	
H1	0.413 (2)	0.1904 (18)	0.1968 (7)	0.0508*	
H2	0.565 (3)	0.291 (2)	0.2768 (8)	0.0723*	
H3	0.49511	0.22482	0.35481	0.0553*	
H4	0.35862	0.07910	0.40130	0.0623*	
H5	0.22913	−0.08592	0.36383	0.0615*	
H6	0.24253	−0.10608	0.27973	0.0505*	
H9	0.33497	−0.03467	0.10984	0.0663*	
H10	0.33127	0.00296	0.02596	0.0746*	
H12	0.43326	0.35179	0.05002	0.0747*	
H13	0.43238	0.31672	0.13369	0.0635*	

### Atomic displacement parameters (Å^2^)

**Table:**

	*U*^11^	*U*^22^	*U*^33^	*U*^12^	*U*^13^	*U*^23^
Cl1	0.1415 (7)	0.1043 (6)	0.0418 (3)	0.0140 (4)	−0.0015 (4)	0.0171 (3)
O1	0.0660 (9)	0.0328 (7)	0.0405 (7)	0.0004 (5)	−0.0077 (6)	−0.0046 (5)
O2	0.0679 (9)	0.0339 (7)	0.0428 (8)	−0.0089 (6)	−0.0031 (6)	−0.0018 (5)
N1	0.0591 (10)	0.0321 (8)	0.0358 (8)	0.0004 (7)	−0.0009 (7)	−0.0028 (6)
C1	0.0373 (9)	0.0326 (9)	0.0366 (9)	0.0072 (7)	0.0002 (7)	−0.0006 (7)
C2	0.0416 (10)	0.0296 (9)	0.0406 (10)	0.0064 (7)	−0.0003 (8)	0.0004 (7)
C3	0.0546 (12)	0.0406 (10)	0.0431 (10)	0.0015 (9)	−0.0044 (9)	−0.0076 (8)
C4	0.0661 (13)	0.0547 (12)	0.0349 (10)	0.0049 (10)	0.0035 (9)	−0.0031 (9)
C5	0.0592 (12)	0.0490 (12)	0.0456 (11)	−0.0030 (9)	0.0109 (9)	0.0038 (8)
C6	0.0441 (11)	0.0371 (10)	0.0450 (10)	−0.0014 (8)	0.0035 (8)	−0.0034 (8)
C7	0.0370 (10)	0.0341 (10)	0.0395 (10)	0.0058 (7)	−0.0015 (7)	−0.0010 (7)
C8	0.0425 (10)	0.0412 (10)	0.0364 (10)	0.0049 (8)	−0.0015 (8)	0.0001 (7)
C9	0.0797 (15)	0.0471 (12)	0.0389 (10)	−0.0058 (10)	0.0010 (10)	−0.0005 (8)
C10	0.0862 (16)	0.0611 (14)	0.0393 (11)	−0.0050 (11)	−0.0014 (10)	−0.0059 (9)
C11	0.0687 (14)	0.0661 (14)	0.0359 (11)	0.0103 (10)	0.0010 (10)	0.0072 (9)
C12	0.0852 (16)	0.0488 (12)	0.0527 (13)	0.0050 (10)	0.0011 (11)	0.0149 (10)
C13	0.0713 (14)	0.0409 (10)	0.0466 (12)	0.0039 (9)	−0.0017 (10)	0.0018 (8)

### Geometric parameters (Å, °)

**Table:**

Cl1—C11	1.738 (2)	C8—C13	1.379 (3)
O1—C7	1.239 (2)	C8—C9	1.382 (3)
O2—C2	1.362 (2)	C9—C10	1.385 (3)
O2—H2	0.82 (2)	C10—C11	1.364 (3)
N1—C8	1.407 (2)	C11—C12	1.370 (3)
N1—C7	1.346 (2)	C12—C13	1.376 (3)
N1—H1	0.84 (2)	C3—H3	0.9300
C1—C6	1.393 (2)	C4—H4	0.9300
C1—C7	1.488 (2)	C5—H5	0.9300
C1—C2	1.404 (2)	C6—H6	0.9300
C2—C3	1.388 (2)	C9—H9	0.9300
C3—C4	1.372 (3)	C10—H10	0.9300
C4—C5	1.378 (3)	C12—H12	0.9300
C5—C6	1.375 (3)	C13—H13	0.9300
			
C2—O2—H2	112.1 (15)	Cl1—C11—C12	119.58 (17)
C7—N1—C8	130.51 (14)	Cl1—C11—C10	120.19 (16)
C8—N1—H1	113.9 (13)	C10—C11—C12	120.23 (18)
C7—N1—H1	115.5 (13)	C11—C12—C13	119.54 (19)
C2—C1—C6	117.65 (15)	C8—C13—C12	120.92 (18)
C2—C1—C7	125.47 (14)	C2—C3—H3	120.00
C6—C1—C7	116.86 (14)	C4—C3—H3	120.00
O2—C2—C3	121.20 (15)	C3—C4—H4	120.00
O2—C2—C1	118.67 (14)	C5—C4—H4	120.00
C1—C2—C3	120.13 (15)	C4—C5—H5	120.00
C2—C3—C4	120.39 (17)	C6—C5—H5	120.00
C3—C4—C5	120.52 (17)	C1—C6—H6	119.00
C4—C5—C6	119.25 (18)	C5—C6—H6	119.00
C1—C6—C5	122.03 (16)	C8—C9—H9	120.00
N1—C7—C1	116.60 (14)	C10—C9—H9	120.00
O1—C7—C1	121.48 (14)	C9—C10—H10	120.00
O1—C7—N1	121.89 (15)	C11—C10—H10	120.00
N1—C8—C9	124.58 (16)	C11—C12—H12	120.00
N1—C8—C13	116.19 (15)	C13—C12—H12	120.00
C9—C8—C13	119.22 (16)	C8—C13—H13	120.00
C8—C9—C10	119.36 (18)	C12—C13—H13	120.00
C9—C10—C11	120.73 (19)		
			
C8—N1—C7—O1	0.3 (3)	C1—C2—C3—C4	−1.4 (3)
C8—N1—C7—C1	−177.67 (16)	C2—C3—C4—C5	0.5 (3)
C7—N1—C8—C9	0.8 (3)	C3—C4—C5—C6	0.8 (3)
C7—N1—C8—C13	−179.92 (18)	C4—C5—C6—C1	−1.2 (3)
C6—C1—C2—O2	−179.53 (14)	N1—C8—C9—C10	179.49 (18)
C6—C1—C2—C3	0.9 (2)	C13—C8—C9—C10	0.2 (3)
C7—C1—C2—O2	−1.1 (2)	N1—C8—C13—C12	179.81 (19)
C7—C1—C2—C3	179.34 (16)	C9—C8—C13—C12	−0.8 (3)
C2—C1—C6—C5	0.4 (2)	C8—C9—C10—C11	0.2 (3)
C7—C1—C6—C5	−178.18 (16)	C9—C10—C11—Cl1	−179.98 (19)
C2—C1—C7—O1	161.80 (16)	C9—C10—C11—C12	0.1 (4)
C2—C1—C7—N1	−20.2 (2)	Cl1—C11—C12—C13	179.35 (18)
C6—C1—C7—O1	−19.8 (2)	C10—C11—C12—C13	−0.7 (4)
C6—C1—C7—N1	158.21 (15)	C11—C12—C13—C8	1.1 (3)
O2—C2—C3—C4	179.11 (18)		

### Hydrogen-bond geometry (Å, °)

**Table:**

*D*—H···*A*	*D*—H	H···*A*	*D*···*A*	*D*—H···*A*
N1—H1···O2	0.84 (2)	1.991 (18)	2.6588 (19)	136.4 (17)
O2—H2···O1^i^	0.82 (2)	1.85 (2)	2.6582 (17)	173 (2)
C9—H9···O1	0.93	2.31	2.895 (2)	121

Symmetry codes: (i) −*x*+1, *y*+1/2, −*z*+1/2.
